# How levosimendan can improve renal function?

**DOI:** 10.1186/s13054-019-2642-z

**Published:** 2019-10-29

**Authors:** Patrick M. Honore, Leonel Barreto Gutierrez, Sebastien Redant, Keitiane Kaefer, Andrea Gallerani, David De Bels

**Affiliations:** ICU Department, Centre Hospitalier Universitaire Brugmann- Brugmann University Hospitals, Place Arthur Van Gehuchtenplein, 4, 1020 Brussels, Belgium

In recent years, several studies have addressed the positive effects of levosimendan upon renal function. We sought to summarize the recent findings in order to understand by which mechanism levosimendan is improving renal function. In acute decompensated heart failure, levosimendan has an immediate renoprotective effect by increasing renal blood flow (RBF) through selective renal arterial and venous vasodilation [1]. In a recent randomized controlled study (RCT) comparing dobutamine and levosimendan in cardio-renal syndrome (CRS), Fedele et al. [2] were able to reproduce the results of Chen et al. [1] by measuring RBF through the renal artery [1, 2]. Fedele et al. and Chen et al. found that levosimendan increased RBF [1, 2]. But increasing RBF alone is not enough. Levosimendan and dobutamine exerted differential effects on glomerular filtration ratio (GFR). Both inotropic agents induced a renal vasodilatory effect accompanied by an increase in RBF, but while dobutamine does not change GFR, levosimendan increases GFR significantly [3]. Levosimendan preferentially causes a vasodilation of the afferent arterioles while dobutamine induces a balanced vasodilation of both afferent and efferent arterioles, thereby increasing RBF, while maintaining a constant glomerular filtration pressure [3]. Obviously, an isolated increase in GFR could jeopardize oxygenation of the medulla, which is sensitive to ischemia, given the highly oxygen-demanding sodium reabsorption process. For levosimendan, however, this is less likely to occur because it causes a balanced increase in GFR and renal oxygen delivery, as shown by the maintained renal oxygen consumption and extraction [4]. Last, a rise in central venous pressure (CVP) is an important predictor of renal dysfunction in heart failure patients [5]. Elevated CVP will increase renal venous backpressure and thus decrease renal perfusion pressure and impair renal function [5]. Compared to dobutamine, levosimendan could further reduce CVP and by this end improve renal function also, but this is not supported by the findings of Lannemyr et al. because both inotropic agents reduced CVP to a similar extent [3]. In conclusion, it is too simplistic to rely upon RBF to expect an improvement in renal function. An increase in RBF has to be accompanied by an increase in GFR but not at the cost of medullary hypoxemia, which is not the case with levosimendan. Effects on the reduction of CVP might also play a role. Finally, luxurious oxygenation of the cortex as induced by dopamine and dobutamine is completely useless for the kidney function, and again, the puzzle of improving renal function is far more complex.

## Data Availability

Not applicable.

## Abbreviations

AKI: Acute kidney injury
RCT: Randomized controlled study
RBF: Renal blood flow
GFR: Glomerular filtration ratio
CVP: Central venous pressure
